# Type 2 diabetes mellitus status in obese patients following sleeve gastrectomy or one anastomosis gastric bypass

**DOI:** 10.1038/s41598-021-83807-8

**Published:** 2021-02-24

**Authors:** Gavriella Zoi Vrakopoulou, Charalampos Theodoropoulos, Vasileios Kalles, George Zografos, Konstantinos Almpanopoulos

**Affiliations:** 1St Propaedeutic Surgical Clinic, Laparoendoscopic Unit, Hippocratio Athens General Hospital, Athens Medical School, National and Kapodistrian University of Athens, 114 Vassilissis Sophias Avenue, 115 27 Athens, Greece

**Keywords:** Diseases, Endocrine system and metabolic diseases, Metabolic disorders, Endocrine system and metabolic diseases, Diabetes

## Abstract

This study aims to compare sleeve gastrectomy (SG) and one anastomosis gastric bypass (OAGB) in terms of remission of type 2 diabetes mellitus (T2DM) in obese patients. All T2DM patients were followed-up for at least 36 months. The primary outcome was remission of T2DM. Secondary endpoints included weight reduction and the procedure’s impact on quality of life. In total, 53/1177 morbidly obese patients who underwent SG (Group A, n = 28) or OAGB (Group B, n = 25) had T2DM. Preoperatively, the mean Body Mass Index (BMI) values were 52.2 ± 8.5 kg/m^2^ and 52.9 ± 10.9 kg/m^2^ for Group A and Group B, respectively. Six patients in Group A were insulin dependent, while 8 were insulin dependent in Group B. After 36 months, diabetes remission was achieved by only 10 patients (35.7%) in Group A. However, in Group B, 22 patients (88%) remained off antidiabetic agents (p < 0.0001), with ΔHbA1c (%) reaching 1.4 ± 1.5% in Group A and 2.7 ± 2.1% in Group B (p = 0.02). Excess weight loss% (%EWL) was again significantly different between the two groups (MA = 79.8 ± 14.5%, MB = 93.3 ± 16.0%, p = 0.003). OAGB is more effective in improving glycaemic control and %EWL, with almost immediate resolution of diabetes, as well as long-term weight loss.

## Introduction

Obesity and its associated comorbidities, including hypertension, stroke, nonalcoholic fatty liver disease (NAFLD), renal failure, dyslipidaemia, and type 2 diabetes mellitus (T2DM), are increasingly important health issues worldwide and pose one of the biggest challenges for healthcare^1,2^. Conservative management of obesity and its related health problems, which includes medical treatment as well as lifestyle modification, can result in a degree of weight loss and improve some of the associated metabolic disorders that are responsible for the development of comorbidities but is unfortunately associated with high failure rates, poor long-term results and minimal overall impact on the cause of the disease itself^3,4^.

In an attempt to achieve long-term weight loss and remission of T2DM, metabolic surgery has emerged as an effective treatment for morbid obesity and obesity-related T2DM^5^. Metabolic surgery has been shown to benefit patients by resulting in sustained weight loss, effectively controlling obesity-related comorbidities such as T2DM, and reducing the risk of cardiovascular events and mortality^6–8^. In the case of glycaemic control, it has been shown that bariatric surgery not only decreases diabetes-related morbidities but can also result in effective and long-term glycaemic control^9^, and there is a growing body of evidence from randomized trials that suggests that compared to optimal medical therapy combined with intensive lifestyle changes, metabolic operations result in superior control of T2DM and metabolic syndrome, including dyslipidaemia, in obese patients^5,10,11^. As a result, obesity surgery is now considered an established therapeutic option for the concurrent treatment of obesity and T2DM^12^.

Established metabolic surgical procedures, such as Roux-en-Y gastric bypass (RYGB) and sleeve gastrectomy (SG), have been demonstrated to improve metabolic disorders and result in significant weight loss^12^. However, the selection of a specific procedure over another remains controversial, as the efficacy of glycaemic control among these different approaches is under scrutiny. Although initial reports from randomized trials claimed that the overall glycaemic control was better for RYGB than for SG in T2DM patients^13^, more recent meta-analyses do not support the superiority of RYGB over SG for the treatment of T2DM^14^.

One anastomosis gastric bypass (OAGB), originally described by Rutledge^15^ and subsequently modified by Carbajo^16^, consists of a technically simpler version of RYGB. Numerous initial studies reported that MGB is a safe and effective alternative to traditional RYGB for the treatment of obesity and T2DM, with the resulting glycaemic control explained by its inherent physiological similarities to classic RYGB^17–20^. As the metabolic results of this approach have now been validated in both the short and long term^21,22^, this approach is gaining popularity worldwide and is now included in the ongoing debate on which metabolic procedure is the most effective in such patients. Therefore, the present study was designed to evaluate and compare the efficacy of SG and OAGB in terms of the control of T2DM in patients with morbid obesity.

## Materials and methods

### Study design and participants

This retrospective study was conducted on a cohort of T2DM patients who underwent LSG (Group A) or OAGB (Group B) from September 2011 to October 2015 in a tertiary hospital that is certified as a centre of Excellence for Bariatric and Metabolic Surgery. The Institutional Review Board (Hippocration General Hospital of Athens, Ref. number 11330) approved this study. The requirement for informed consent was waived for all the participants by the IRB.

Data from a prospectively maintained database of the Bariatric and Metabolic Surgical Department in our institution were reviewed. Patients were eligible for inclusion in this study if they (1) had type 2 diabetes according to the ADA guideline criteria^23^, (2) underwent SG or OAGB as a primary surgery, and (3) had completed at least 36 months of follow-up. Patients who (1) suffered type 1 diabetes mellitus, (2) had a history of previous bariatric surgery, or (3) were not compliant with the department’s follow-up schedule were excluded.

All patients complied with the guidelines of the American Society of Metabolic and Bariatric Surgery, which require a body mass index [BMI] ≥ 40 kg/m^2^ or a BMI ≥ 35 kg/m^2^ and one or more obesity-related comorbidities to undergo any kind of bariatric surgery. To define T2DM, we used the 2019 ADA guideline criteria, i.e., glycosylated haemoglobin (HbA1C) ≥ 6.5%, fasting plasma glucose of 126 mg/dL, or 2-h postload plasmatic glucose ≥ 200 mg/dL during an oral glucose tolerance test (OGTT)^23^. Hence, we considered patients to have diabetes if they were receiving antidiabetic agents. For diabetes remission, we used the Position Statement from the Association of British Clinical Diabetologists (ABCD) and the Primary Care Diabetes Society (PCDS), i.e., achievement of glycaemia below the threshold currently used for diagnosis of type 2 diabetes that is sustained for a minimum period of 6 months while the patient discontinued all glucose-lowering therapies^24^.

The operating surgeon had a one-on-one consultation with each patient to evaluate the nutritional habits (big meals, binge eaters, sweet eaters, etc.) and physical status and explained in detail the appropriate procedure, its perioperative risks, and the postoperative and follow-up course. Subsequently, all patients were evaluated by a multidisciplinary team that included nutritionists, endocrinologists, and psychologists.

Smokers were excluded from receiving OAGB due to an increased risk for an anastomotic ulcer^25^. Patient demographic characteristics, weight, comorbidities, HbA1c, antidiabetic medications, and changes in or discontinuation of treatment were recorded at baseline; 1, 3, 6, 12, 18, and 24 months postoperatively; and annually thereafter. All patients were followed-up for at least 36 months. The primary outcome was remission of T2DM (HbA1c < 6.5% without glycaemic therapy). Secondary measures included T2DM improvement, weight progression, % total weight loss (%TWL), % excess weight loss (%EWL), and lifestyle changes related to glucose levels.

### Preoperative assessment

Preoperative evaluation included a detailed medical history and physical examination by a bariatric surgeon. All patients underwent oesophagogastroduodenoscopy, *Helicobacter pylori* detection tests, electrocardiography, abdominal ultrasound, and laboratory tests (including estimation of fasting plasma glucose [FPG], glycated haemoglobin [HbA1C], thyroid hormones [T3, T4, TSH], and vitamin D).

Current smokers or patients who quit smoking less than 6 months prior were excluded from receiving OAGB. For the rest of the patients, the type of surgery was decided after oesophagogastroduodenoscopy and according to the patient’s eating habits. For binge and sweet eaters, OAGB was the treatment of choice, while for overeaters, we considered SG the more adequate surgical option.

Hence, all patients had a psychological evaluation before surgery or psychiatric reevaluation for those under medical treatment to reassure that they were able to understand and cope with postoperative lifestyle changes.

For patients with a BMI > 50, additional preoperative evaluation was needed, including pulmonary function testing and triplex ultrasound testing for the heart, veins, and arteries of the lower extremities. Patients were asked to follow a low-calorie diet one month prior to surgery under nutritionist guidance.

### Surgical technique

#### LSG

All SG procedures were performed with a strictly standardized procedure comprising a series of key steps^26^. An optical trocar was used to enter the peritoneal cavity. After identifying the pylorus, dissection of the greater omentum began 5–7 cm cephalad. Subsequently, we entered the lesser sac and ligated all the branches of the gastroepiploic vessels at the level of entrance into the greater curvature. Dissection was continued until the gastric fundus was fully mobilized. After insertion of a 38 cm Bougie, the stomach was divided along the lesser curvature using a stapling device. Drainage is placed only in cases of revision but not routinely.

#### OAGB

The surgical technique for OAGB is described in great detail throughout the surgical literature^20,21^, and the tailored version that we performed in our department was described in our previously published study^27^.

However, it should be emphasized that in our approach, the length of the bypassed biliopancreatic loop depends on the patient’s preoperative BMI. After locating the Treitz ligament, the surgeon will bypass either 200, 250, or 300 cm of the small bowel for preoperative BMIs of < 50, 50–60, or > 60, respectively. Hence, our latest practice involves the measurement of the total bowel length to reassure that we bypass equal to or less than 1/3 of it.

All OAGB procedures were performed by a single surgeon in the first Propaedeutic Surgical Clinic, Laparoendoscopic and Bariatric Unit (Center of Excellence for Bariatric and Metabolic Surgery) at the Hippocration General Hospital of Athens, Greece.

### Early postoperative period

Postoperatively, patients were allowed clear fluids and mobilization 4–5 h following surgery, according to the Guidelines for Perioperative Care in Bariatric Surgery of the ERAS Society^28^. A routine gastrografin swallow study was not performed prior to feeding. Laboratory tests included haemoglobin, haematocrit, white blood cells and neutrophils on the same day plus C-reactive protein (CRP) for the 1st and 3rd days and every second day for 2 weeks postoperatively in an outpatient clinic or a nonhospital laboratory. A CRP value greater than 200 mg/L is interpreted as pathognomonic of leak, as described in a previously published study^29^.

Patients with an uneventful postoperative course were discharged on the 1st or 2nd p.o day. Patients were instructed by our nutritionist to undergo a stepwise advance from a liquid diet (1st p.o week) to pureed (2nd p.o week) and to soft foods (4th p.o week). After 1 or 2 weeks on soft meals, most patients begin introducing some solid food and could progress to consuming all solids as tolerated. Patients were also prescribed and advised to consume high caloric (≈ 1.5 kcal/mL) and high protein (≈ 10 g/100 mL) energy drinks targeting a total protein intake of 60–120 g daily^32^. Furthermore, patients were placed on PPIs for the first 6–12 months, depending on their medical history and symptoms. Antithrombotic prophylaxis with low-molecular-weight heparin (LMWH) was advised for the first 20 days postoperatively.

### Follow-up

Patients were followed up on the 1st, 3rd, 6th, 12th, 18th, and 24th months p.o. and then once annually thereafter. In this study, we included only patients who had been followed-up for a minimum period of 36 months. A multidisciplinary team consisting of a bariatric surgeon, a nutritionist, an endocrinologist, and a psychologist monitored patients at each follow-up visit. Weight variations, changes in medical treatment, episodes of hypoglycaemia, number of meals/day, fitness activity, and blood test results (including full blood count, serum levels of glucose, HbA1c, cholesterol, iron and ferritin, calcium and vitamin D, albumin, vitamins and trace minerals) were noted. Hence, all patients underwent surveillance upper GI endoscopy and abdominal ultrasound on an annual basis.

### Statistical analysis

We conducted statistical analysis using the Statistical Package for the Social Sciences (IBM, SPSS, version 23.0). For normally distributed data, t test was used. Thus, we performed a Pearson product–moment correlation coefficient to detect any possible correlations between patient values and chi-square for independence to determine a relationship between two categorical variables. For all statistical tests, a p value < 0.05 was considered significant.

### Ethical approval

All procedures performed in studies involving human participants were conducted in accordance with the ethical standards of the institutional and/or national research committee and the 1964 Declaration of Helsinki and its later amendments or comparable ethical standards. The requirement for informed consent was waived for all the participants by the IRB.

## Results

Since April 2009, a total of 1177 obese individuals had undergone metabolic surgery in our department. Among them, 895 patients underwent SG and 282 OAGB. A postoperative interval of at least 36 months was attained for 473 and 115 patients, respectively. Of these, at the time of the operation, 28 SG patients (15.1%) and 25 OAGB patients (26.6%) suffered from T2DM and were using either oral or parenteral antidiabetic medication.

### Patient characteristics

The sex distribution was 12 men (42.9%) and 16 women (57.1%) in the SG group and 10 men (40.0%) and 15 women (60.0%) in the OAGB group (Table 1). The average age at operation was 45.9 ± 7.5 years and 46.6 ± 7.8 years in the SG and OAGB groups, respectively (Table 1). No statistically significant difference was detected between the two patient subgroups regarding the aforementioned parameters.

**Table 1:** Obese T2DM patient characteristics.

**N**	
SG, n (%)	28 (52.8%)
OAGB, n (%)	25 (47.2%)
**Age**	
SG, mean ± SD (range) (years)	45.9 ± 7.5 (23–62)
OAGB, mean ± SD (range) (years)	46.6 ± 7.8 (32–65)
**Sex**	
SG, men, n (%)/women, n (%)	12 (42.9)/16 (57.1)
OAGB, men, n (%)/women, n (%)	10 (40.0)/15 (60.0)

*T2DM* type 2 diabetes mellitus, *SG* sleeve gastrectomy, *OAGB* one anastomosis gastric bypass, *SD* standard deviation.

The two groups also did not differ significantly regarding the preoperative weight-associated parameters, namely, total body weight (TBW), excess body weight (EBW), and body mass index (BMI) (Table 2). EBW was calculated as the weight surplus in relation to the ideal body weight (IBW), which is the weight that corresponds to the individual patient’s height for a BMI of 25 kg/m^2^.

**Table 2:** Obese T2DM patient preoperative somatometric values.

**IBW**	
SG, mean ± SD (range) (kg)	73.7 ± 9.7 (57.8–99.0)
OAGB, mean ± SD (range) (kg)	73.4 ± 8.5 (60.1–95.1)
**Preoperative TBW**	
SG, mean ± SD (range) (kg)	153.8 ± 30.4 (92.0–215.0)
OAGB, mean ± SD (range) (kg)	154.9 ± 37.4 (111.0–245.0)
**Preoperative EBW**	
SG, mean ± SD (range) (kg)	80.1 ± 26.4 (34.2–146.9)
OAGB, mean ± SD (range) (kg)	81.5 ± 34.0 (39.4–164.0)
**Preoperative BMI**	
SG, mean ± SD (range) (kg/m^2^)	52.2 ± 8.6 (38.0–79.0)
OAGB, mean ± SD (range) (kg/m^2^)	52.7 ± 10.8 (36.6–79.7)

*IBW* ideal body weight, *TBW* total body weight, *EBW* excess body weight, *BMI* body mass index, *SG* sleeve gastrectomy, *OAGB* one anastomosis gastric bypass, *SD* standard deviation.

### Patients’ diabetes history

A positive family history of DM was present in 19 patients (67.9%) in the SG group and 16 (64.0%) in the OAGB group. No significant difference was found concerning patient age at diagnosis or the duration of DM, with a mean disease duration of 63.4 ± 52.7 months for the SG group and 69.1 ± 47.8 months for the OAGB group (Table 3). SG patients were prescribed an average of 1.4 antidiabetic agents, whereas 1.6 agents were prescribed for each OAGB patient. Concerning glycaemic control, the mean glycated haemoglobin (HbA1c) values were 7.1% and 7.8% in the SG and OAGB groups, respectively (Table 3). These differences were also non-significant. For adequate glycaemic control, insulin remedies were required to a significantly greater degree in the OAGB group [(8 patients (32.0%)] than in the SG group [6 patients (21.4%)] (p = 0.046).

**Table 3:** Obese T2DM patients’ history of DM.

**Positive family history**	
SG, *n* (%)	19 (67.9)
OAGB, *n* (%)	16 (64.0)
**Age at diagnosis**	
SG, mean ± SD (range) (years)	40.6 ± 7.1 (20.0–54.0)
OAGB, mean ± SD (range) (years)	40.8 ± 6.7 (30.0–54.0)
**Duration**	
SG, mean ± SD (range) (months)	63.4 ± 52.7 (12.0–156.0)
OAGB, mean ± SD (range) (months)	69.1 ± 47.8 (10.0–180.0)
**Number of antidiabetic agents**	
SG, mean ± SD (range) (agents)	1.4 ± 0.8 (1–3)
OAGB, mean ± SD (range) (agents)	1.6 ± 0.8 (1–4)
**IDDM**	
SG, *n* (%)	6 (21.4)
OAGB, *n* (%)	8 (32.0)
**Preoperative HbA1c (%)**	
SG, mean ± SD (range)	7.1 ± 1.2 (6.0–10.7)
OAGB, mean ± SD (range)	7.8 ± 2.0 (5.9–13.0)

*T2DM* type 2 diabetes mellitus, *IDDM* insulin dependent diabetes mellitus, *HbA1c*(%) glycated haemohemoglobin, *SG* sleeve gastrectomy, *OAGB* one anastomosis gastric bypass, *SD* standard deviation.

### Early postoperative complications

In our institution, overall, there is a 6.2% complication rate among patients undergoing SG and a 4.3% complication rate for OAGB patients in the first 30 days postoperatively. Considering major complications (Clavien–Dindo grade ≥ III), specifically, the respective rates for SG and OAGB were 1.2% and 1.8%, respectively. Among the patients included in the study, 2 SG patients (7.1%) and 1 OAGB patient (4%) (p = 1.000) suffered a complication in the early postoperative period. These complications included one case of atelectasis and one case of intraabdominal abscess in the SG group and a case of minor postoperative bleeding in the OAGB group. All of these complications were managed conservatively.

### Long-term results

Complete follow-up data were available for all patients included in the study. Substantial weight loss was achieved for patients in both subgroups, with the mean BMI at 36 months postoperatively being 33.8 ± 6.5 kg/m^2^ for the SG group and 31.4 ± 6.5 kg/m^2^ for the OAGB group. A significantly higher percentage excess weight loss (%EWL) was observed in the OAGB group than in the SG group, with relative values of 79.7 ± 14.5% (SG) and 98.2 ± 29.0% (OAGB), respectively [t(50) = − 3.182, p = 0.003] (Table 4). Excess weight loss was calculated by comparing weight loss at 36 months postoperatively to the preoperative EBW. The proportion of patients with a %EWL greater than 50% designates the success rate as 96.4% for SG and 100.0% for OAGB in this time interval.

**Table 4:** Body weight-associated parameters 36 months postoperatively.

**Weight loss**	
SG, mean ± SD (range) (kg)	54.3 ± 20.3 (21.0 – 100.0)
OAGB, mean ± SD (range) (kg)	62.1 ± 31.4 (10.0 – 143.0)
**BMI**	
SG, mean ± SD (range) (kg/m^2^)	33.8 ± 6.5 (25.1 – 58.8)
OAGB, mean ± SD (range) (kg/m^2^)	31.4 ± 6.2 (19.5 – 52.5)
**BMI Loss**	
SG, mean ± SD (range) (kg/m^2^)	18.4 ± 6.4 (7.0 – 30.9)
OAGB, mean ± SD (range) (kg/m^2^)	21.3 ± 10.4 (3.5 – 50.7)
**%EWL**	
SG, mean ± SD (range) (%)	79.7 ± 14.5 (49.7 – 108.1)
OAGB, mean ± SD (range) (%)	98.2 ± 29.0 (73.4 – 215.1)
**Operation success**	
SG, *n* (%)	27 (96.4)
OAGB, *n* (%)	25 (100.0)

*BMI* body mass index, *EWL* excess weight loss, *SG* sleeve gastrectomy, *OAGB* one anastomosis gastric bypass, *SD* standard deviation.

**Figure 1:**
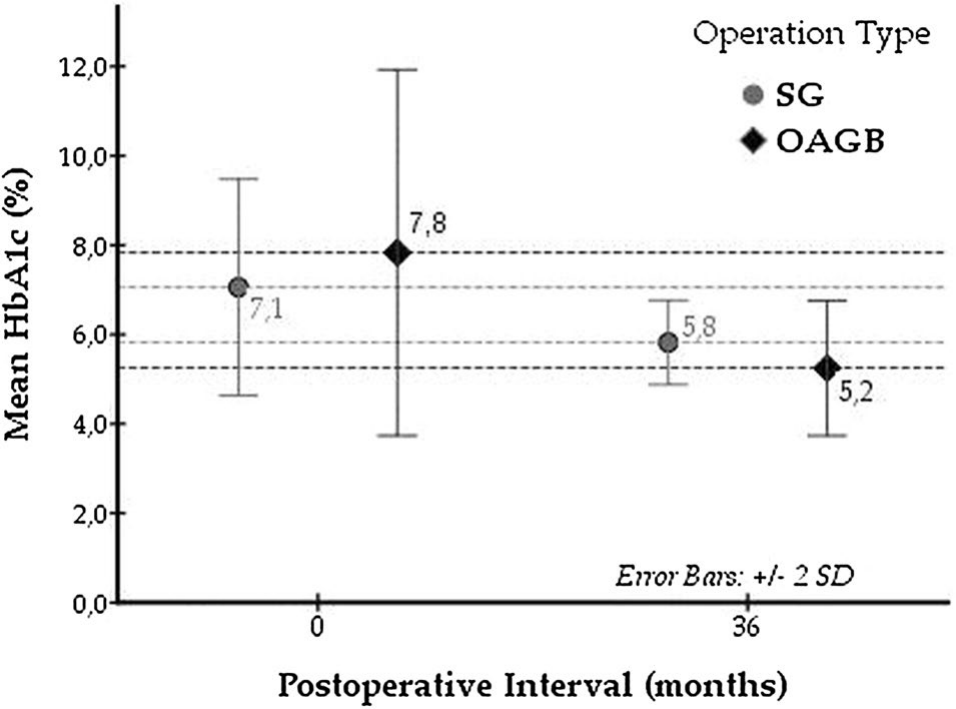
Mean absolute values, as well as the produced differences, of HbA1c (%) levels in the SG and OAGB groups at the time of the operation and 36 months postoperatively.

A significantly greater reduction in HbA1c was observed in patients who underwent OAGB than in those who underwent SG. The average HbA1c value for the SG patients at 36 months postoperatively was 5.8%,% while the corresponding absolute value for the OAGB group was 5.2%, producing a mean decrease of 2.7% and an average difference of 1.4% [t(44) = − 2.419, p = 0.020] (Fig. 1).

**Figure 2:**
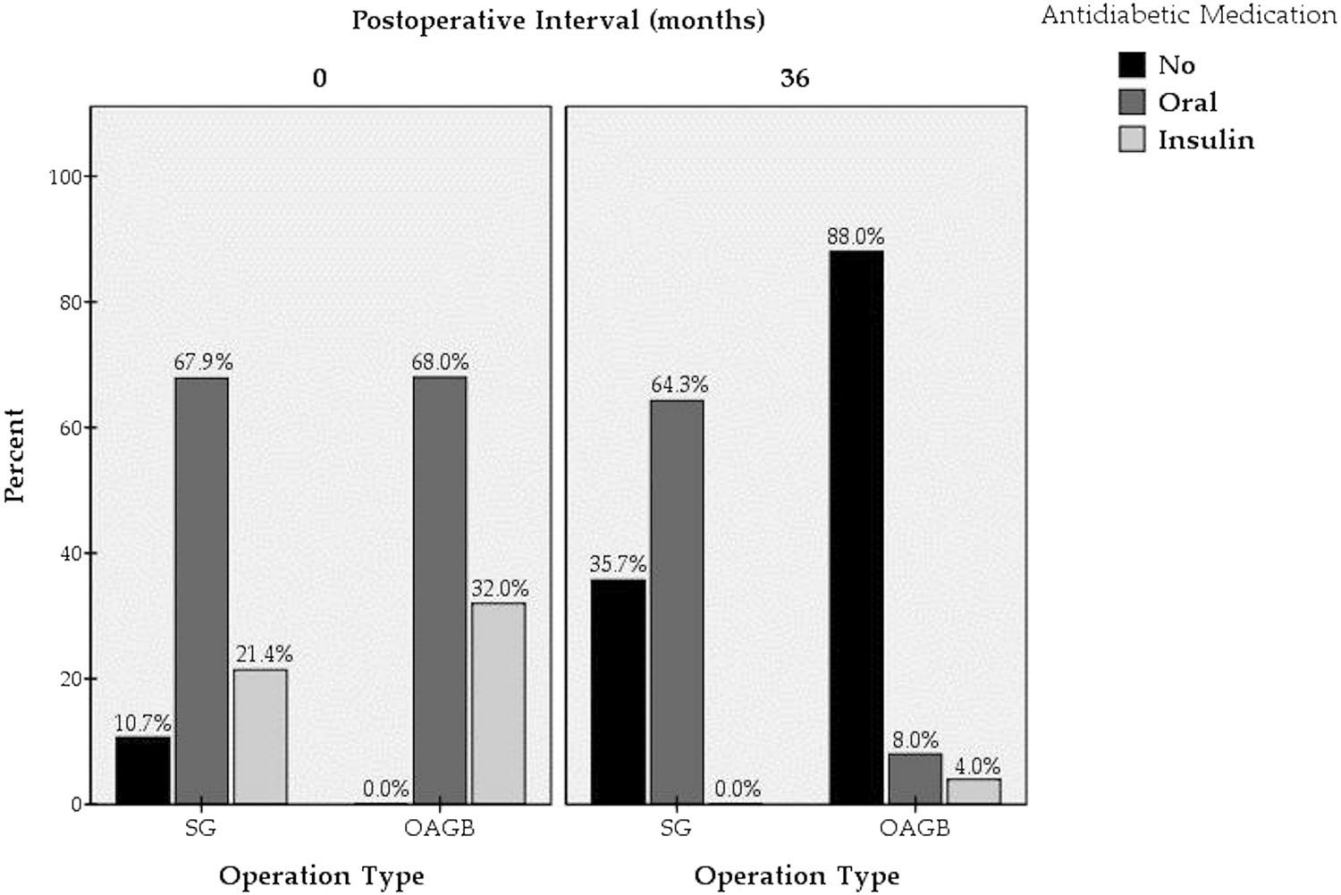
Antidiabetic medication required for DM control for both patient subgroups at the time of operation and 36 months postoperatively.

Furthermore, a more favourable diabetes management outcome was evidenced by the significantly greater number of OAGB patients who achieved euglycaemia while weaning from all antidiabetic agents. Thus, only 35.7% of SG patients needed no medication, while this was true for 88% of OAGB patients (U = 77.0, p < 0.001) (Fig. 2). Of note, even though treatment discontinuation was not achieved for all patients, downregulation of the required treatment was achieved for both patient subgroups. Moreover, the majority of patients were able to discontinue their antidiabetic medication, even before being discharged from the hospital.

Regarding lifestyle modifications imposed following the bariatric procedure, patients in both groups had, on average, 3–4 meals per day, with only a minority in either group attending physical exercise programmes on a regular basis.

Finally, 7 SG patients (25.0%) and 8 OAGB patients (32.0%) admitted experiencing hypoglycaemic episodes during the last month, with no significant difference between the two groups. The majority of patients complained of an average of two episodes per week. Further insight, however, revealed that lack of compliance with the daily meal schedule preceded episodes of hypoglycaemia.

## Discussion

Bariatric surgery has proven its efficacy and benefits in terms of long-term results and improvements in patient quality of life. Success rates regarding weight loss and resolution of comorbidities differ between bariatric procedures, i.e., sleeve gastrectomy, Roux-en-Y gastric bypass, one anastomosis gastric bypass, etc.^30^. However, these surgical modalities were only recently added to the official diabetes treatment algorithms^31^ since many of them have been shown to be highly effective in the treatment of T2DM^32,33^.

Thus, it is now well accepted that not only restriction and malabsorption but also other mechanisms related to visceral signals and weight-independent factors such as hormones, the nervous system, bile acids, and the gut microbiota, as well as the interactions between them, determine the outcome of metabolic surgery^34^. This fact makes all available procedures, even strictly restrictive ones, candidates for the “fight” against diabetes in bariatric patients. However, the selection of a specific procedure over another remains controversial, as the efficacy of glycaemic control among different approaches is under scrutiny. In this study, we examined the effect of OAGB versus that of SG in T2DM patients with similar preoperative characteristics.

In terms of %EWL, our study is consistent with previous studies reporting results in favour of OAGB. The OAGB group achieved a significantly higher %EWL of approximately 98 ± 29.0%, whereas the SG group reached 79.7 ± 14.5% at 36 months postoperatively [t (50) = − 3.182, p = 0.003]. However, our rates are superior to those reported by Shivakumar et al.^35^, who attained a %EWL of 66.48% ± 15.72% in the OAGB group at the end of the 3rd year of follow-up, while in the LSG group, the %EWL was 61.15% ± 25.27%.

Furthermore, in our study, 88% of patients who underwent OAGB remained off antidiabetic agents 3 years after surgery, reflecting their status of normoglycaemia, in comparison to the significantly lower 35.7% in the SG group. This difference was statistically significant (p < 0.001), and although studies report that diabetes remission or recurrence follows the trend of %EWL and weight regain, our results indicate nonproportionally low remission rates for SG patients. In a previous study, Musella et al.^22^ compared diabetes remission rates between OAGB and SG patients 1 year postoperatively and reported a significant advantage of OAGB, with rates of 85.4% versus 65.9%, respectively. Hence, a randomized control trial from Shivakumar et al.^35^ demonstrated diabetes remission in 89.13% of MGB-OAGB patients and 81.82% of LSG patients 3 years after surgery, without reaching statistical significance. Thus, Kular et al.^36^ reported the 5-year T2DM remission rate in 2014, and the Mini Gastric Bypass group was superior to the SG group (92% versus 81%, *p* < 0.05).

It appears that the rates of diabetes remission associated with OAGB outweigh those associated with SG, and this effect is durable over a long period of time. Our significantly lower rate of diabetes remission 3 years after SG compared to those reported in previous studies could be a result of bias associated with the limited sample size and the retrospective nature of our analysis. Furthermore, there has been substantially less homogeneity in the literature with regard to the criteria defining diabetes remission or restoration of normoglycaemia in T2DM patients.

With regard to HbA1c change, our data seem to imply a greater reduction in the OAGB group, with a mean decrease of 2.7% compared to SG, where the average difference was 1.4% [t (44) = − 2.419, p = 0.020]. This seems to be unrelated to the BMI reduction, which in this moderate number of patients was not statistically significant. However, our results are consistent with those of the European survey conducted by Musella et al.^26^ comparing the %change vs. baseline values for HbA1c in relation to BMI reduction, where no significant correlation was found (ΔHbA1c 0.4 for MGB/OAGB; ΔHbA1c 0.1 for SG).

Attention must be paid to the fact that even though some patients did not achieve diabetes remission or had recurrence during the postoperative period, the number or dosage of agents needed to achieve normoglycaemia was reduced.

This is a retrospective study conducted with prospectively collected data, and there is an ongoing follow-up process. As such, we cannot offer a safe conclusion unless a larger number of patients is analysed, and prospective studies are needed to further support our preliminary results.

However, despite the moderate number of patients included in this retrospective analysis, the homogeneity of the preoperative characteristics and the high follow-up rate with the complete set of data available for each patient for the 3-year follow-up period give credibility to our study.

## Conclusions

In our study, OAGB proved to be a more efficient method for treating T2DM obese patients over a 3-year period of time. Patients submitted to OAGB achieved diabetes remission at a significantly higher rate than their LSG counterparts. However, for both groups, a reduction in dosage or number of antidiabetic agents needed for euglycaemia was recorded. This result is not in accordance with %EWL rates, as the analysis demonstrated no statistically significant difference between the two groups at the 3-year follow-up. This indicates that weight loss alone cannot overcome the metabolic effect of the bypass and opposes the supporters of LSG submitting T2DM obese patients to a purely restrictive procedure. A longer follow-up time and a larger sample size are needed to confirm our preliminary results.

However, our study adds to the literature, as it is primarily designed to assess the effectiveness of these two methods on diabetes control. Thus, concerning the major difficulties of recruiting and following up patients for RCTs, it is important for the results of different certified bariatric surgery centres to be reported.

### Limitations

Our study has several limitations. First, the limited sample size and the medium follow-up duration were major weaknesses. Our results should therefore be regarded as preliminary. Second, this was a retrospective study of medical records, and selection bias cannot be ruled out.
